# A Treatise on the Diseases and Injuries of the Spinal Nerves

**Published:** 1834-10-01

**Authors:** 


### A Treatise on the Diseases and Injuries of the Spinal Nerves. By *Joseph Swan*, &c. &c.

[Third and concluding Article.]

The business of the critic can scarcely, perhaps, be dignified with the appellation of an art. His office in the intellectual world is analogous to that of the "maitre de cuisine," in the animal, and probably the hungry author will apply to both the same opprobrious adage, with reference to the origin of the viands and the cook.

The combinations of the latter have exercised the ingenuity and gratified the inclinations of every age, since the æra of Prometheus, the thief of fire. A mighty nation is justly proud of its incontestable pre-eminence in the direction of the kitchen, and the modern descendants of the warlike subjects of Clovis and Charlemagne, are less likely to be excelled in the *science* of gastronomy, than in that of war.

If the gratification of the palate is accounted the peculiar excellence of a great people, and if the professors of the pleasing art are appreciated and rewarded by the noble and the wise,* the occupation of the critic should be

* Louis Eustace Ude is said to rejoice in his box at the opera.

equally honoured and remunerated. But we fear that since the period when the Roman Knight established the dependence of the members on the belly, the latter has exercised an undisputed sovereignty. If a celebrated culinary physician were asked which he deemed the most glorious—the prescription of physic or preparation of food, we have little doubt that the palm would be awarded to the latter. Were a committee of Crockford's appointed to decide on the respective utility of Ude and of Gifford, the experienced better would probably wager on the triumph of the former.

We will not attempt to balance the merits of cookery and criticism, yet we must contend that as natural genius and assiduous study are universally admitted to be necessary to form the accomplished cook, some portion, at least, of reflection and experience are requisite to constitute the dexterous critic. The world would seem to disregard this reasonable postulate, and even Byron has believed that critics could be ready-made. The conductor of a Journal has frequent opportunities of lamenting the prevalence of this monstrous error. Correspondents amerce him in his pocket for amicable communications and obliging hints on the best way of managing his own affairs. His Majesty's post is the unexpensive medium of advice, which if taken, would have probably the effect intended—the reduction of the advised party to the pleasant situation described in the fable of the Man, his Son, and his Ass.

Did the critic cater for a single taste, a moderate experience might enable him to satisfy it. But when it is his office to replenish mouths of different dimensions, and to tickle palates of different susceptibilities, the man who gives only one dish and one sauce may possibly sometimes find them rejected.

We have offered these remarks in consequence of receiving some friendly communications on the best mode of editing this Journal. One gentleman has suggested a few very long articles, another has counselled a multitude of short ones. If these critical amateurs were confronted with each other, we should probably be amused by an interesting argument on taste. One excellent monitor has hinted that we indulge in scurrility and impiety. He dates his letter from the Modern Athens, the native seat of toddy and religion.* Perhaps he was offended at our *blasphemous* denunciation of the midnight processions, and the pulpit alarums, for which Scotland was distinguished in the time of the cholera. To his injurious accusations we disdain reply. Whilst the sober Scotsman has been shocked at our scurrility, and would measure our step by the solemn gait of the tragic muse, a Southron well-wisher has lamented our sedateness, and deplored our want of spirit.

The ingenuous exposition of contradictory complaints, will usually convince the candid judge of their general injustice. The reader will perceive, that the Editor of an extensively circulated Journal is under the necessity of trusting to his own judgment, and of following the course which experience, or tact, induce him to believe most widely useful, and most generally approved. By such a standard he is actually tried, and he cannot be rewarded or amerced at the arbitrary tribunal of individual inclination.

---

* "Rum and true religion."—*Byron.*

This, which may be looked on as a species of apology, has appeared to us to be required on offering a *third* notice of the book before us. Nothing can be further from our wish or our intention, than the paltry attempt to spread a continuous article through successive numbers of this Review, for the purpose of provoking or sustaining curiosity. Such devices are too shallow, too contemptible to be entertained by any but the venal conductors of some penny trash. When the subject admits of natural divisions it is frequently convenient to adopt them. In the instance of the present work, the second notice formed a large component part of an article upon the nerves, which occupied 28 of our closely printed pages. Had the notice been completed in that article, it must either have been slurred and in all respects imperfect, or its bulk would have exceeded all reasonable limits.

It may not be thought impertinent if we make a few remarks on the subject of medical reviewing. Three classes of books are presented by the authors with equal confidence to the notice and approbation of the critic. The first is extremely good, the second is extremely bad, and the third is neither. No doubt can exist with respect to the treatment of the first class—the temper and tact of the reviewer will regulate his severity or forbearance towards the second—but the real difficulty is experienced in the management of the third. If too much space is allotted to a work of the latter description, the public is dissatisfied—if too little, the author and the author's friends are discontented.

Were the good works numerous, the reviewer's labour would be light—were the bad ones universal, his difficulties would be inconsiderable—the predominance of productions too good to be neglected, too indifferent to deserve the dedication of much attention, is that which perplexes the experienced critic, and ruins the incautious one.

Being in some degree Utilitarians, and thinking that the true objects of a medical review are the advantage and instruction of that portion of the profession, who are not possessed of the leisure for perusing or the means for purchasing those works which display and which contribute to the advance of science, we have always leaned to the side of analysis rather than of criticism. The former is more mechanical, more laborious, and more adapted to the purpose of diffusing information—the latter is more shewy, and less useful. We conceive that, in general, a lengthened abstract of one valuable work, is preferable to a meagre notice of a dozen. The latter is little superior to a catalogue—it provokes an appetite which it cannot satisfy, and insults the understanding by its impudent imitation of substantial aliment. Yet the paucity of original books of real value must compel the critic to abandon the hope of an exclusive plan; and the nature of the circumstances renders it advisable to combine the method of presenting a full account of good works, with the expedient of offering brief criticisms, or rapid sketches of inferior ones.

The medical public are perhaps not aware of the labour undergone by the conductors of an Analytical Review. A principal part is experienced, where probably it would scarcely be expected to exist, in finding matter adapted for their operations. Many a volume which is brought to their alembic, is composed of materials too coarse and too valueless to be analyzed, or too imponderable to be secured. The fatiguing process of examination is merely productive of disappointment and rejection. The Editors of this Journal have laboured at least long, if they have not laboured well. They possess experience, and may claim the useful merit of industry. They have had little reason to complain of the public, and they trust that the public will find no just occasion to complain of them.

From this preliminary digression, we are recalled to the subject immediately before us. Five chapters of the work of Mr. Swan, consisting of more than one hundred and thirty pages await examination. The subjects of those chapters are—Diseased Appearances in the Spinal Canal—Injuries of the Spinal Chord—Diseases of the Nerves of the Senses—Diseases of the Sympathetic Nerve—and Tetanus.

### DISEASED APPEARANCES IN THE SPINAL CHORD.

This chapter consists of the particulars of four cases. We fear that it can lay no claims to the possession of a complete or scientific arrangement, in fact that is no more than the expression of four insulated facts. The object with which they are brought forward is praiseworthy:—to add to the existing catalogue of authentic morbid appearances discovered in the vertebral canal. We shall briefly report the principal particulars.

CASE 1. *Hydrocephalus—thickening of the Spinal Arachnoid.*

A boy, eight months old, was seized with vomiting and fever, succeeded by opisthotonos, convulsive motions of all the muscles of the body, and insensibility, with dilated pupils. In a fortnight or three weeks after the commencement of the attack, the patient died.

On dissection, Mr. Swan found the fontanelle wider than it should have been, and a great quantity of fluid in the ventricles of the brain. On opening the spinal canal, three patches of a reddish substance, about the surface of a horse-bean, lay on the dura mater, about the beginning of the dorsal vertebræ, but he could not decide whether these were owing to disease. From the level of the first dorsal vertebra, the spinal arachnoid was thickened and opaque, and had coagulable lymph effused on it as far as the beginning of the cauda equina. The pia mater appeared to have been involved in the disease. The morbid appearances were confined to the posterior part of the chord.

We suppose that it is fair to consider the opisthotonos, if not the convulsions, as special symptoms produced by the condition of the spinal arachnoid.

*Case* 2. In a man, aged 25, who was hanged for murder, the spinal arachnoid had several patches of a cartilaginous substance on it. In each of the choroid plexuses was a yellowish tumour, about the size of a large pea. There was much fluid in the ventricles. The man had been confined in the gaol for many weeks, and during the whole time was in perfect health.

Mr. Swan observes, that similar appearances in cases of tetanus cannot fairly be considered as the causes of it. We imagine that little doubt can be entertained of the frequency of mistakes respecting morbid changes and appearances of the spinal marrow and its membranes.

Case 3.—*Epilepsy—Cartilaginous State of the Spinal Dura Mater and Arachnoid.*

A man, aged 45, was admitted into the Lincoln County Hospital, on account of epileptic fits, under which he had laboured, at intervals, for seven years. He died in the hospital, from an attack of erysipelas.

On dissection, the longitudinal sinus was found to terminate in the left lateral sinus, and there was a communication between the two lateral sinuses very little larger than would admit the blunt end of a probe. The right lateral sinus appeared to be a continuation of the torcular Herophili. This arrangement must, apparently, have tended to retard the return of the blood. There was some fluid between the dura mater and arachnoid membrane of the brain, and the whole arachnoid covering the convolutions was very opaque and much thickened. Some small patches of cartilaginous matter were observed on it.

On the inner surface of the spinal dura mater was one small scale of cartilaginous matter. The arachnoid membrane adhered very much to the dura mater within the cervical vertebræ, and there were many adhesions through its whole extent, especially on the posterior part. There were several scales of cartilaginous matter on it, the same as in the preceding case.

Case 4. *Reddish Substances on the outside of the Dura Mater.*

In the first of these cases, Mr. Swan alluded to some patches of a reddish substance, which lay on the outside of the dura mater. He has frequently observed similar appearances in different parts of the spinal canal, and more especially in the inferior portion. He cannot, therefore, at present, consider them the consequences of disease. In dissecting the body of a man whose lungs were very much diseased, he found such an appearance covering all the posterior part of the dura mater of the spinal canal, to a very unusual extent. There was a fluid very much like pus at several of the inferior spinal holes. On opening the dura mater, many adhesions were found between it and the arachnoid membrane, especially that part contained within the cervical vertebræ; some of these adhesions were long, like threads. Some bloody fluid was found in the inferior part of the sheath.

Such are the contents of the chapter on "diseased appearances of the spinal canal." It is evident that the title is too bold. It should have been worded, with more truth and more hesitation—on *some* of the diseased appearances in the canal.

We think we hinted in a former article that Mr. Swan is not happy in his theoretic ventures. The reader may be rather tempted to smile, than to be convinced, when Mr. Swan informs him, that he has known hiccup very troublesome in affections of the chest, and that he conceives it may depend upon disease communicated to the phrenic nerve.

The thirteenth chapter is devoted to injuries of the spinal chord.

### On Injuries of the Spinal Chord.

This consists, like the former chapter, of narratives of cases, with the interposition of a few remarks, chiefly of a speculative character. It is a subject of regret that Mr. Swan should display two material defects in the construction of this work—want of method in the arrangement, and prolixity in the details. A little reflection might convince him, that the simple transfer of particulars, whether trivial or important, from the private case-book to the published volume, is an easy but an unsatisfactory mode of compilation. It requires little judgment, and still less genius to scrape together the notes of all the cases we have seen of a particular description, and denominate them a chapter, or section, or essay on the malady of which they are promiscuous specimens. We are advocates for the publication of cases, because we conceive that a philosophical work should approach as much as possible to the character of demonstration, and that opinions should be little more than the simple and obvious expression of facts. But a superficial knowledge of the laws of logic and of reasoning is sufficient to inform the medical writer, that irrelevant details and unimportant particulars, obscure what they do not illustrate, and fatigue rather than convince. To employ the language of the logician, the object of the exact and perspicuous writer should be to discover and enunciate the *essential mode* of the subject upon which he treats, and to strip it of those accidental attributes which encumber and conceal its essence. The expansive tendency of the facts of Mr. Swan is great, for one case is diffused through sixteen pages of his work.

We shall select those facts which appear to merit attention. The following appears to be an instance of concussion of the spinal marrow, or, at least, of an injury not amounting to much actual physical lesion.

*Case.* " A man, about thirty years of age, fell from a waggon on his back: he immediately had violent pain in his back, with convulsions of its muscles, attended with most excruciating pain; for a minute or two he would be comparatively easy, and then the convulsions would return with great violence. He was bled, took a large dose of laudanum, and a mixture with sulphate of magnesia dissolved in peppermint water, to which some ether was added: his back was frequently fomented and rubbed with anodyne liniment. The pain continued in this way violent for about twelve hours, and then gradually abated: at the end of twenty-four hours he had passed no urine, and as the bladder was very much distended, and he had much pain from it, his water was drawn off: he continued after this to expel his urine whenever he pleased: the pain kept gradually diminishing, and in abour a week all symptoms of complaint, except general weakness, left him." 225.

In the next case the injury was greater, and probably some alteration was produced in the organization of the medulla.

*Case.* A man, æt. 33, whilst on the ground, had his head forced violently forward. He immediately became insensible, and as it was supposed that his neck was dislocated, a man immediately held him fast between his knees, and having his hands fixed under the lower jaw, drew up the head forcibly, and the patient became instantly sensible. But all the parts below the neck were now discovered to be paralytic, the right side more completely than the left, sensation and motion being lost on the former, while imperfect sensibility was retained upon the latter. On the next day, he was seen by Mr, Barton, of Market Raisin, who found considerable tumefaction about the lowest cervical vertebra, pain in that situation, a paralytic state of the body, and fever. By venæsection, and other antiphlogistic remedies, he was relieved. Although no distortion of the spine could be detected, pain and increased numbness were occasioned by certain movements of the arms, and by deep lateral pressure on the vertebræ. It appeared to Mr. B. that there was a fracture connected with the right transverse process.

In January, 1820 (the accident had happened in the preceding September), he was admitted into the County Hospital. He had the perfect feeling in every part of the body, except the extremities of the thumbs and fingers, which felt very numb; the muscles below the injury were as completely paralytic as they ever had been, but he could distinguish whatever touched him: he complained of a pain in his head, which had continued ever since the accident; he complained likewise of a very great pain in the right shoulder: there were some enlargement and tenderness on pressure about the last cervical vertebra: he appeared in good health as far as the functions of the viscera were concerned. Mr. Swan made a seton on each side of the affected vertebra. Soon after they began to discharge, he regained some power over the muscles. At the end of a month, he could just stand by himself, and walk, when steadied and supported by another. He had some use of the left arm, but very little of the right. We need not pursue the details. We need only state that he was discharged from the hospital in June, with such use of his lower extremities, as to be enabled to walk to a considerable distance. The use of his arms was also much improved, and he was entirely free from pain when they were moved. He was in perfect health. All enlargement and tenderness about the vertebra had disappeared.

This case will afford an apportunity for making a remark, with reference to the treatment of injuries of the spine. We think that the general occurrence of inflammatory action of the membranes or the substance of the spinal marrow, as a consequence of injury, has not been sufficiently attended to. The profession are anxiously alive to the supervention of inflammation of the brain, after blows upon the head—they endeavour to prevent it by precautionary depletion—watch every symptom, in order to arrive at a ready recognition of it—and, when it has occurred, they treat it with activity and boldness. Yet when similar injuries have been inflicted on the spine, the patient is frequently allowed to take his chance, with little assistance from the surgeon or physician. He is placed upon his back, his diet is restrained, and his bowels may be regulated, but, so far as our opportunities of observing and of reading have extended, the catalogue of remedies most commonly embraces few further, nor any more potent items.

Yet, if cases of this description are accurately studied, it will be found that the symptoms commonly characteristic of inflammatory action are usually established at an earlier or a later period, subsequent to the infliction of the injury. The pulse increases in frequency and force, the skin grows warm, the secretions are altered, pain may be felt in the seat of the injury, and the parts supplied by nerves from the medulla opposite and below the affected part, display the conditions which vascular excitement of the medulla, or pressure on it from effusion, might be thought to be respectively likely to produce. It would be foreign to our purpose to pursue this subject at the present moment. We have merely thrown out the hint, which observant surgeons will discover, we believe, to be founded in truth. We have reason to hope that Mr. Brodie will communicate the results of his observation and his experience on this subject to the public, at no distant period, and we are much mistaken if he does not present a more scientific and exact account of the symptoms, the consequences, and the treatment of injuries of the spinal column, than has yet been offered to the profession.

Mr. Swan's observations on this subject are so judicious, that we willingly extract them.

"There are three occurrences always to be feared after an injury of the spine, any one of which sooner or later is destructive of life, and therefore their prevention ought to occupy seriously the mind of every surgeon. The occurrences I allude to are inflammation spreading to the medulla or its membranes; disease of the bladder; and mortification of the lower part of the back and nates.

When the spine is injured, the same changes take place as in injuries of other parts of the body. Inflammation, in a greater or less degree, is set up, and if the injury is below the part that supplies nerves to organs immediately necessary for the maintenance of life, it is the inflammation, I believe, which causes death, when it happens very soon after the accident. It becomes, therefore, necessary to prevent inflammation of the chord and its membranes, by general and topical bleeding; indeed the same care ought to be taken as in injuries of the head. The diet should be of the mildest kind, and an absolute state of rest in a recumbent position should be enjoined. It is not enough for the symptoms immediately ensuing on the accident to be removed, but attention to diet ought for some time to be adhered to, and every exertion of the body avoided, and especially riding in a carriage over rough roads. Should pain arise in the injured part, blood should be taken from it by leeches or cupping; or should numbness or any other symptom denoting impaired functions of the chord be complained of, blood may be taken away in the same manner; and if the patient is not relieved, setons or issues should be made near the part. In mentioning setons and issues I would by no means recommend their being made immediately over a fractured vertebra, unless some weeks have elapsed since the accident, as issues especially may communicate with the fracture, and make it a compound one, thus causing irreparable injury.

The urine should be drawn off twice or three times in twenty-four hours; and if the bladder be insensible, so much greater ought to be the care taken in using the catheter, for an injury may be easily done; and when ulceration has begun in a part deprived of the influence of the brain, nature seems to have but little power in controlling it.

Mortification of the parts below the injury must be prevented by keeping them very clean and dry, and washing them with a spirituous embrocation, as brandy, &c." 251.

In some cases, small general bleedings are productive of advantage. Of course their employment must be regulated by the general condition of the patient, and the actual character of the symptoms. In a case of severe injury of the dorsal portion of the spine, which was admitted into St. George's Hospital, repeated bleedings, to the amount of four or five ounces at a time, were highly useful. The blood displayed the usual characters of inflammation, as the patient evinced its ordinary symptoms. He had perfect paralysis of all the parts below the seat of injury. He gradually recovered, and we met him about twelve months ago, walking without the aid of a stick in the street.

Mr. Swan appears to deprecate, rather than to approve, the proposal to trephine the spine in cases of fracture with depression. He plausibly, indeed fairly, urges the difficulty of determining the nature and amount of injury, and observes that, were this as easy as it is impracticable, the propriety and safety of the operation would continue doubtful. The first argument is so weighty, that we fancy the operation will continue a subject of speculative reasoning, rather than one of practical application.

There are several omissions in Mr. Swan's notice of the effects of injury upon the spine. He has not alluded to, or has faintly glanced at, the alterations of the temperature of the body—the disturbance of the urinary secretions and organs—the variations in the sensibility and mobility of the parts below the seat of injury—the condition of priapism—and the structural changes which occur in the medulla. These are severally matters of interest and of importance to the scientific and the practical surgeon.

### Diseases of the Nerves of the Senses.

These form the contents of the fourteenth chapter. Mr. Swan first considers disease of the olfactory nerves; but we perceive little in his observations to detain us. The only remark to which we think it necessary to allude, is in reference to the perception of unpleasant odours, when no apparent external cause for them exists: and no obvious alterations of the nerves, of the apparatus of smell, or, indeed, of any part of the body whatever, is present to account for them. He compares this unnatural condition of smell, to what is observed when morbid impressions are made on the optic nerve, spots of various colours and shapes being seen, as though they had a real and substantive existence. When this "morbid action" (the term may be exceptionable) of the nerves of smell is set up, the functions of the stomach and viscera connected with them are, as Mr. Swan assures us, frequently deranged, and if these are restored to a healthy state, the disorder will generally cease.

From diseases of the olfactory, Mr. Swan proceeds to those of the optic nerves. He subjoins some description of amaurosis, which we may omit. But he offers some observations, dictated by experience, on the treatment of "dizziness."

"When it comes on frequently, I have found it very difficult to cure by the means usually employed for that purpose. As I have in several obstinate cases removed it, I think it may not be useless to relate what has occurred to me on the subject.

When a person has become subject to dizziness, though he may in the first instance have been relieved by bleeding, yet should the complaint soon return, and especially if the body is much debilitated, a farther loss of blood will not only not relieve it, but will, on the contrary increase it. In many cases the usual remedies may be employed with advantage; but there are others in which the complaint is continued from habit, which must be interrupted by every thing that can improve the general health; and, with this view, I have several times given, with success, from half a drachm to a drachm of powdered bark every four hours; at the same time allowing a generous diet, with the use of wine. I have often thought malt liquor prejudicial.

Instead of dizziness, or accompanied by it, some people will have a very confused sensation in the head, attended with debility of body, restlessness, palpitations of the heart, and mental irritation, almost amounting to insanity; which I have known to be cured by the same means." 260.

Of course, dizziness is a symptom of several conditions of the cerebral or other systems. The inexperienced surgeon or physician must not rashly follow Mr. Swan's advice. Proper care must be exercised in discriminating cases.

When children, says our author, are affected with a disordered state of the digestive organs, or worms, squinting is frequently produced, in consequence of the impaired action of the abductor muscle, through an affection of its nerve produced by its communication with the sympathetic.

We will not follow Mr. Swan through his devious pursuit of the functions of the lenticular ganglion. His reasoning is ingenious, and his suppositions may be just.

In treating of diseases of the gustatory nerves, Mr. Swan observes, and he quotes a case in point from Sir Everard Home, that these nerves are sometimes violently bruised between the teeth, and that loss of the sense of taste has been the consequence. In a woman who was bitten on the tip of the tongue by a wasp, the part became swollen and very painful, and she lost the gustatory sense for a week. Mr. Swan also alludes to the well-known circumstance, that taste is rendered imperfect, by obstruction to the passage of air through the nostrils. But we cannot admit his explanation to be a happy one. He supposes that the absence of taste is produced by the air, which should pass through the nose, being carried between the tongue and the palate, and keeping those parts too much asunder. A simple experiment may convince any one that this is not a necessary consequence of obstruction of the nasal passage, and a more philosophical rationale of the circumstance may be found, in the consideration of the compound nature of taste. It is proved that this consists of the impression produced by sapid substances on the tongue, and, perhaps, upon the palate; and of the odour of the substance, or some sensible quality, appreciated in the nares. The obstruction of the passage of air through the latter prevents the development of this species of sense, and impairs, in that ratio, the general function of taste.

Mr. Swan occupies twenty-two pages with diseases of the auditory nerves, or rather with some observations upon deafness. These are not arranged with sufficient precision to enable us to lay their substance before our readers, in the space which the pressure of other articles permits to dedicate to this. Yet we will endeavour to select the most novel or peculiar.

He first relates a case in which, after an injury of the head, and the flow of much blood from the right ear, accompanied with insensibility, permanent deafness of that ear ensued. He supposes that the auditory nerve was greatly injured or destroyed.

The characters of nervous deafness next occupy his attention.

"The functions of the auditory nerves may be impaired so as to produce deafness. When this is the case, the patient finds he cannot hear sounds that he was accustomed to, and is at the same time tormented with various noises, which are compared to the undulations of the sounds of bells, humming of bees, waterfalls, &c, and if the complaint increases, he becomes so deaf as not to hear at all without the greatest difficulty."

"The noises attending nervous deafness, likewise attend diseases of the external auditory meatus, as when it is filled with hardened cerumen, and likewise when there is a diseased state of the membrane lining the meatus. When there are noises with a loss of the sense, and the external auditory meatus and the membrane of the tympanum have every appearance of being in a healthy state, if the patient stops his nose and mouth and blows downwards, and feels that peculiar sensation which every one does when the Eustachian tubes are perfect, and if a watch cannot be heard except very faintly, when it is in contact with the head, face, neck, or teeth, we may be certain that the disease is in the nerve." 269.

We fear we know too little of the real functions of the different component parts of the auditory apparatus, to enable us to acquiesce, without misgivings, in the foregoing positive annunciation. We conceive that, when the argument is fairly weighed, its value amounts to little more than this, that in the cases described by Mr. Swan, the external meatus and the membrane of the tympanum are probably not concerned in the production of the deafness, and that it is certainly not dependent on obstruction of the Eustachian tube. Beyond this, we see no certainty nor safety.

The treatment recommended by Mr. Swan, in cases of this nature, consists of mercurial purgation, with antiphlogistic regimen, and the application of blisters behind the ears. The patient should be bled, when symptoms of fulness of the cranial circulation are present.

Mr. Swan has some ideas, or rather some suspicions, on the action of the branches of the glosso-pharyngeal nerve, distributed upon the tympanum, which, although more fanciful than likely to produce conviction, may not be unworthy of the notice of the scientific reader.

"In tracing the tympanine branch of the glosso-pharyngeal nerve, which has been so particularly described by Jacobson, much of its distribution may be seen on the transparent membrane lining the tympanum when this part is perfectly sound, but when it is diseased a very considerable difficulty is experienced. In an attempt to trace this nerve in the head of an old woman, the membrane lining the tympanum was not only thickened, but there was at the same time some roughness of the bone. In the head of a man, who had a suppurating node on the forehead, and whose posterior nostrils were stopped up by adhesions of the soft palate, this membrane was also thickened; the spheno-palatine ganglion was very considerably enlarged. In the dissection of the head of a very young woman, the Schneiderian membrane, covering the inferior turbinated bone of the left nostril, adhered very considerably to that of the septum, so that a very little passage was left for the air; there was a perforation in the membrane of the tympanum of the same side, and purulent matter was contained in each tympanum. The membrane lining the tympanum was so much thickened, that the nerves could not be observed.

I believe deafness does not so often depend on a disease of the auditory nerve as has been supposed, but much more frequently on an inflammatory action attacking the membrane lining the tympanum, and involving these small branches of the tympanine nerve. There are very few deaf people who cannot hear music or singing, or who cannot hear conversation, whilst they are in a carriage in motion. But it is not so with those who are nearly blind, for when the optic nerve is paralysed, no light, or any modification of it, can produce perfect sight, and it must be the same with the auditory nerves with respect to sound. I will not deny that a very strong light may enable a person who has a slight degree of vision to see some objects almost in the same manner as a very deaf person hears with a speaking trumpet. I believe, therefore, that deafness depends very frequently on the inflammatory action which has impaired these minute branches of the glosso-pharyngeal nerve, distributed on the tympanum; and although many of the noises may depend on the disordered functions of the auditory nerve, I nevertheless think they may arise, too, from these small branches of the glosso-pharyngeal, and their communication with the sympathetic in the carotic canal." 271.

A less ingenious man than Mr. Swan might regard the chronic inflammatory thickening of the tympanum as sufficient, *per se*, to account for the phenomena, independent of any particular alteration of a branch, so small as to be almost imperceptible, of a nerve whose office continues problematical. The microscopic eye of the physiological anatomist observes, with attention, objects that escape the vague glance of common men.

Mr. Swan alludes to the occurrence of deafness in consequence of exposure to very loud sounds, when the ear is unprepared to receive them. He relates the case of a Captain Norton. We conjecture, from the circumstances, that the Captain had been exposed to the sound of artillery, in some of those actions which seldom redound to aught but the honour of our gallant sailors. We will let our hero tell his own story in his own way.

"In reply to Mr. Swan's questions, Captain Norton begs to state, that blood only came from the left ear, being in a right line towards the percussion; yet the singing or intense buzzing was equal in both ears—sometimes like the chirping of ten thousand sparrows, and at others a monstrous hissing. The first day Captain Norton could not hear any thing, and the second day could only hear a person who placed his mouth close to the ear, and spoke in a modulated tone; could not hear the high notes of a fife; could not distinguish the tune; he could not hear the boatswain's call (whistle.)

Some four or five weeks afterwards Captain Norton was again in action, and was close to the muzzle of a gun when fired, and the report most completely restored his hearing for two days, when the singing again commenced. Captain Norton is of opinion that his hearing is as acute as ever, and it is only the singing which confuses sounds; for when one person only is speaking, he can hear him tolerably well; but when three or four are speaking at the same time, he cannot distinguish any one: he can hear a distant gun or bell as well as any one." 274,

Mr. Swan imagines, what, indeed, is more than probable, that in this and similar instances, the membrane of the tympanum is ruptured. He supposes that, where particular sounds can be heard, but words cannot be sufficiently distinguished, in a conversation carried on by several persons, (occurrences sufficiently common in deafness), the reason may probably be found in the membrane of the tympanum and that of the round fenestra not acting in unison. We leave this supposition for the assent or investigation of our readers. We may state, however, that Mr. Swan is laudably actuated by the desire to encourage the patient and practitioner with the hope, that deafness may depend more frequently on affections of the tympanum, a part within our reach, than on distant and inaccessible conditions of the auditory nerve. He thinks it probable, that many of those affections have their origin in inflammatory affections of the tympanum, and that numerous instances of deafness would be prevented, were the surgeon more judicious and more active in treating the ear-aches and similar complaints of early life.

Mr. Swan brings forward some curious facts, in order to establish a singular hypothesis:—That the portio dura of the seventh pair of nerves contributes to the sense of hearing. Let our readers listen to Mr. Swan's argument.

"When the ears are stopped, and a watch is brought in contact with any part of the head, face, teeth, or neck; or if a stick, water, &c. be interposed between any of these parts and the watch, the sound will be heard as well as when the ears are open.

It has been supposed that the sound is mechanically conveyed through the flesh and bone in the same way it is through a macerated bone, piece of wood, &c.; but if it were so, it must be heard always when the auditory nerve is perfect, at whatever part of the head, face, &c. the watch is applied, but this is not the case. When the hearing through the external meatus has been perfect, and there has been no apparent alteration in the structure of the head, face, &c., I have seen many who could hear from only one of these parts, and several who could not hear from any of them.

"If I stop my ears and rest my chin on the petrous portion of the temporal bone in a macerated skull, and place my watch in contact with any part of the skull, I can hear the sound perfectly. I saw a boy who was born deaf and dumb, but had been taught to speak, and when a watch touched the left side of his face he could hear it, but when it touched any part of the right side he could not in the least.

A man who was recovering from an illness had become so deaf of the left ear that he could just hear my watch when put very near it: he heard perfectly of the right ear. I desired he would stop his ears until he could not hear my watch when put nearly in contact with them; I then let it touch the left side of the face, &c.; he just heard it, but when I let it touch the right side, he heard it distinctly.

If sound is conveyed mechanically through the flesh and bone, what in these two cases should hinder it from being heard distinctly, when the watch touched either side of the face, any more than in the macerated skull?

If sound is not conveyed mechanically through the head, face, &c., it must be through some other medium, and that I believe to be the facial nerve of the seventh pair and some other nerves connected with it.

On dissecting the seventh pair of nerves in man, I find at the bottom of the internal auditory meatus a communication between the auditory and facial nerves.

In sheep I have observed the same communication.

In fishes several nerves, that have a communication with the auditory nerve, are spread on the skin over the whole head.

If we consider how the facial nerve is connected by nervous substance with the auditory, its extraordinary course, its receiving the branch of the vidian nerve and the chord of the tympanum, and, when it has got out of the stylo-mastoid foramen, its great expansion, I think we may conclude that it was made to serve some greater purpose than has hitherto been ascribed to it." 281.

The idea is ingenious, but we do not completely understand its applicability to the facts. Why should a boy, born deaf and dumb, hear the ticking of a watch placed in contact with the left side of his face, and remain insensible to the same sound, when any part of the right side of the face was touched? Was the portio-dura on the latter side paralyzed? Probably Mr. Swan could dispel the doubts that cling to the sceptical mind of the critic. Our author, indeed, pursues the supposition through several pages, and through a lengthened series of experiments performed on an individual born deaf and dumb. Whilst we beg to refer the inquisitive reader to the work of Mr. Swan, we must take the liberty of hinting our belief that he has not perfectly made out his case. Yet we cannot enter the arena of argument, and perhaps it is not just to assume the rights of victory, when prudence or necessity compel us to decline the fight.

### On Diseases of the Sympathetic Nerve.

The experienced reviewer has witnessed the decline and fall of numerous attempts to determine and distinguish the diseases of the sympathetic nerve. The genius of Bichât almost grasped its physiology, yet the doubt which is gathering, rather than subsiding, on this interesting question, may shew how inadequate are the efforts of even a master's mind, to decide by reasoning, subjects that can be proved only by direct demonstration and induction.

Mr. Swan commences by shewing that Bichât did not hold such exclusive opinions as have been supposed, on the want of sensibility in the sympathetic nerve. It requires, indeed, but a moment's reflection to shew the apparent incompatibility of such a notion, with the various sensations experienced in health and in disease, in the parts to which the sympathetic is distributed. Yet in estimating the functions of this nerve, or rather system, the reasoner and the experimenter are met by an obvious and a startling difficulty. The nerve in question is so connected and involved with the cerebral and spinal nerves, that it seems impossible to decide on the respective parts which they play in the production of phenomena.

Mr. Swan relates a case in which the temporal bone was destroyed, and was removed by the process of exfoliation. The sympathetic nerve on that side was ulcerated, but so many other nerves were affected, and such various mischief was produced, that we cannot conceive the case to prove much in a physiological sense.

Mr. Swan relates the particulars of two experiments, performed with the view of determining the functions of the sympathetic. In the first experiment, a portion of the nerve was cut out on each side of the neck of a rabbit. No particular effect was produced. On killing the animal, three months afterwards, the extremities of the nerves were found to be united by new filaments. The second experiment was similar to the former, and so was the result. What they prove must be deemed of a negative quality.

Mr. Swan conducted some other experiments, for the purpose of ascertaining the manner in which inflammation of a part of the body produces constitutional irritation and fever. We will offer an abstract of an experiment of this description.

"At a quarter before 10, a. m., of Jan. 11, 1825, a piece of gamboge weighing 76 grains, was inserted in a wound between the shoulders in the back of a very large dog. He soon after began to be very uneasy. In the evening he lapped much water, and afterwards vomited. He purged in the night.

12th, at seven, a. m. He lies as if he were almost dead. He sometimes moves and howls, and then appears in a state of stumor. He cannot stand. At half-past nine he was in the same state. Whilst I was observing him, there was a violent spasm of all the muscles of the body, which lasted about a minute; he vomited at the same time. He died about eleven." 300.

The dog was examined at one, p. m.

All the ganglia of the sympathetic nerves were very highly inflamed.

The pia mater of the brain was rather more vascular than natural. There was a small quantity of serous fluid about the base of the brain. There was a very small quantity of serous fluid in the sheath formed by the spinal dura mater. The axillary nerves and the nervus vagus were more vascular than usual.

The liver and spleen displayed an increased vascularity. The stomach contained some half-digested food which had been eaten before the experiment was made. Its villous coat near the pylorus was very red; in the other parts there were numerous black spots, at each of which there was an ulcer, and the black part appeared to be coagulated blood. The villous coat of the small intestines had a very high degree of redness, and was ulcerated in many places. The mucous coat of the large intestines was more red than usual.

The wound in the back was closed; when its lips were separated, a quantity of gamboge, dissolved in the serum, flowed out, but some of it had insinuated itself amongst the cellular membrane, nearly as far as the elbow. There was a most violent and extensive inflammation of the cellular membrane, and a corresponding effusion of serum.

It will be noticed that we have Mr. Swan's authority for stating, that all the ganglia of the sympathetic nerves were highly inflamed. It is true that Mr. Swan does not enter into satisfactory particulars, nor inform us what appearances constituted inflammation. Yet Mr. Swan would seem to be satisfied, not only of the reality of the inflammatory state in this instance, but even of its general occurrence, in cases where constitutional irritation follows as a consequence of partial inflammation. He says:—

"After every accident in which the constitution sympathises with the injured part, I believe, the ganglia of the sympathetic nerves become irritated, and the functions of the parts supplied by their branches are disturbed in consequence. The action of the heart is increased in proportion to this degree of irritation in them, so long as it continues moderate." 304.

This general enunciation of opinion and of facts may spare us the necessity of special allusion to our author's cases. We may introduce another observation:—that, in consumption, and complaints attended by hectic fever, Mr. Swan has not found an increased vascularity of the ganglion of the sympathetic nerves; but that, should acute inflammation supervene, the symptoms change, and an increased action in the ganglia is the consequence. We will not pursue the subject of vascularity of the ganglia any further. It may fairly be presumed that Mr. Swan, acquainted as he is with the anatomy and the pathology of the nervous system, would not heedlessly mistake a casual or an insignificant appearance for a morbid change of decided character and important nature. His observations are therefore entitled to the attention of those who are inclined and enabled to follow up the investigation. We may add Mr. Swan's reasons for concluding that the appearance he describes is really inflammatory.

"Although I am well convinced, from numerous dissections, that the ganglia of the sympathetic nerves have a great vascularity, induced only by several medicines and diseases, yet some are inclined to think that this appearance is present in a state of health. In answer to this opinion, I beg to observe, that I examined an executed subject immediately after it was cut down, and found the ganglia of a pearly appearance, and free from any mark of vessels carrying red blood. I have found one ganglion very red from a number of vessels filled with blood, and the corresponding one nearly white; and I have so often observed this difference, and in such a marked degree in the same subject, as to leave no more doubt in my mind of its being the effect of inflammation, or something bordering on that state, than of similar appearances constituting the inflamed conjunctiva of one eye, and the uninflamed state of the other. If a judgment be formed from the appearances presented by an injected subject, it will be erroneous, for it is always impossible to speak decidedly of the natural degree of vascularity of any part which has undergone such a preparation. The ganglia may be made red by injection, and so may the conjunctiva of the eye; we may therefore fairly conclude, that if the injection fills numerous vessels of the eye, which could not be observed during life, that this appearance is equally foreign to the ganglia during health." 322.

The question may be asked—why such various symptoms should exist during life, when inflammation of the sympathetic ganglia is discovered after death? This reasonable query receives no satisfactory reply from Mr. Swan. Contenting himself, for the reasons he has stated, with believing that the appearances mentioned are inflammatory, he abandons the attempt at solving the difficulties which surround the production of symptoms. We cannot quit the subject without the observation, that, obscure as the pathology of the sympathetic is, we think that it is worthy of more minute attention than it seems to have hitherto received.

The last chapter of this work is occupied with the perplexing subject of Tetanus.

### ON TETANUS.

The researches of our author on the morbid changes which invade the sympathetic nerve, appear to have enabled him to triumph, in some degree, over the obscurity that shrouds from the common ken, the pathology of tetanus. The obstinate sceptic may hint that the dubious and hypothetical foundation is unable to support any stable edifice. Let the candid reader attend to Mr. Swan.

"With a view of removing as much as possible this obscurity, I have been induced to inquire how the body is usually affected after accidents. From that inquiry I have been led to state, that when a severe injury has been received, the ganglia of the sympathetic nerves become irritated, and consequently the parts to which they distribute nerves. When the constitution is healthy, I believe the irritation of the ganglia goes off in a few days, and then the parts supplied by them return to a state of quietude and again perform their healthy functions.

When the ganglia of the sympathetic nerves have been thus affected, and the irritation has subsided, an unhealthy action in the wound may excite a fresh irritation in them. Or even if the wound be healed, the passions, improper food, and other causes, may continue, reproduce, or increase the disordered state of the organs receiving nerves from the ganglia, and thereby excite a fresh irritation in them.

When the ganglia of the sympathetic nerves have once been in a state of irritation, I believe they are very susceptible of its renewal. When they have become again irritated, it may be readily conceived that the irritation, modified by the confinement during the healing of the wound, or by some disordered viscus, may be very different, and by this change made much more formidable in its consequences; and it may therefore be readily conceived, how in this state it may be communicated to many of the cerebral and all the spinal nerves, and from these to the spinal chord; thus producing tetanic spasms, varying according to the part of the sympathetic nerve most affected, as well as the extent and complexity of the irritation." 326.

We fear there is some ground for doubt and disbelief in the foregoing explanation of cause and consequence. The potential mood is liberally used in describing the possible affections of the ganglia, and important circumstances are *believed* and *conceived,* when the rigorous reasoner might expect them to be proved.

Mr. Swan relates four cases and one experiment in order to display the affection of the sympathetic ganglia in tetanic cases. In the instance of the experiment, tetanus was produced, and a rabbit was destroyed by the alkoholic extract of nux vomica. On examining the animal after death, all the ganglia of the sympathetic nerves, and especially those of the right side, had a considerable redness. The lumbar ganglia were less red than any of the rest. The par vagum was unusually vascular. The axillary plexus, especially of the right side, was rather more vascular than usual, and the same was observed respecting the sciatic nerves. The pia mater of the brain and spinal chord was very vascular; so were the absorbent glands, and the villous coat of the stomach. The lungs were very purple, and there was an increase of vascularity on the outside of the aorta.

The particulars of one case will afford an idea of the morbid appearances on which Mr. Swan has founded his opinions.

*Case.* The patient died on the sixth day from the commencement of the tetanus. He had been treated by turpentine and calomel and opium.

On examination after death, the spinal sheath was found to contain a small quantity of limpid fluid. Many adhesions were found between the loose arachnoid membrane and that lining the dura mater. On the loose arachnoid membrane there were a few small spots of cartilaginous matter. The veins of the pia mater were much loaded with blood. The spinal chord was divided in many places, and appeared perfectly healthy; but in the beginning of the dorsal portion there was a spot of coagulated blood, of the size of a small pin's head, in the midst of the medullary substance. There was an effusion of bloody serum between the arachnoid membrane and the pia mater of the brain, at the situation of the squamous portion of each temporal bone. The veins were turgid, but there was not any diseased appearance in the brain.

The visceral alterations were not numerous nor important. They consisted of congestion of the lungs—a patch of coagulable lymph upon the heart—vascularity of the mucous membrane of the stomach and the bladder. Blood was effused into the substance of each psoas muscle.

The par vagum in the neck was quite healthy, but at the root of the right lung the nerve had an increased vascularity, which did not exist in that of the opposite side.

There was an enlargement and a greatly increased vascularity of all the ganglia of the sympathetic nerves in the chest, and also of the semilunar ganglia; in several of those in the abdomen, the same appearance existed, only in a less degree, but in some there was neither the least redness nor enlargement.

As a summary of Mr. Swan's opinions, we may state that, although he does not intend to assert that tetanus is a specific complaint, entirely seated in the ganglia of the sympathetic; he conceives that those ganglia are the important parts of the nervous system, to which the first irritation tends, and from which it proceeds to the remainder of that system.

Mr. Swan relates some cases in which tetanus was evidently the consequence of injury of a nerve. We will rapidly enumerate their chief particulars.

1. A patient was operated on in the old manner for popliteal aneurism, by Sir William Blizard. The aneurismal sac sloughed out, and the healing process was advancing, when Sir William endeavoured to extend the limb from the flexed position in which it had remained since the performance of the operation. The patient suddenly expressed agonizing pain, and within a few hours was seized with tetanus, of which she died. On dissection, the cutaneous nerve, which accompanies the lesser saphena vein, appeared to have been divided in the operation, and the inferior portion having become attached to the contiguous parts, had been stretched in the attempt to extend the limb. Such was the explanation of the occurrence of the pain and succeeding tetanus, which appeared most consistent with the facts.

2. In a case of tetanus which occurred to Dr. Hennen, the radial nerve was somewhat thickened, and a splinter of bone was sticking in it.

3. A case is mentioned by Dupuytren, in which a patient died from tetanus, after a small wound in the forearm, occasioned by a blow from a coach-whip. On examining the cicatrix, M. Dupuytren was greatly astonished to find a portion of the whip enveloped in the very substance of the cubital nerve.

3. Larrey relates three cases where it was produced by an injury of the larger nerves. In the first, the anterior crural and sciatic nerves had been injured by a ball; in the second, the median nerve had been tied with the brachial artery; and in the third, the nerves had been tied in amputation of the leg.

4. Mr. Swan possesses the thumb of a man in which there was a partial division of one of the dorsal branches of the radial nerve, the semilunar ganglia were also highly vascular.

5. To these cases related by our author we may add two others which occurred at St. George's Hospital. The first was that of a man who was stuck with a pitch-fork in the outside of the leg. He died of tetanus. On examination of the limb, it was found that the pitchfork had wounded the peroneal nerve, and that a sort of nodule, the result of inflammatory action and thickening, was developed in its substance. The second case was that of a boy, who slightly wounded the sole of his foot, in climbing over some iron railings. He died of tetanus. The spike was found to have injured the internal plantar nerve, which displayed such a thickening as that described in the preceding instance.

It is possible that, in other cases, a superficial examination of the injured part may have failed to observe the implication of the larger nerves or their branches; it is probable that in many instances, the smaller twigs may be more or less involved in the cicatrix.

The frequent failure of amputation of the wounded part has destroyed the confidence of surgeons in the measure. Yet cases have been published, in which it would seem to have produced a cure. Division of a nerve succeeded in a case detailed by Dr. Murray, in the Medical Gazette for February, 1833. The wound was in the sole of the foot, and had been inflicted by a rusty nail. The posterior tibial nerve was divided, and the patient recovered.

Larrey relates two cases, in which incisions were beneficial. They are practically interesting.

"The first was that of a man who was struck by a ball, which crossed the right arm, and wounded the biceps and coraco-brachial muscles, and the radial and internal cutaneous nerves. On the eighth day he began to have great pain; and it was wished to divide a bridge left by the wound, in which were found some branches of the internal cutaneous nerve, but the patient refused to have it done. The next day his local pains were very acute; he had convulsive motions of the hand and fore-arm, heat in the whole system, and locked jaw; he was very restless, and in continual agitation. The rapid progress of the symptoms determined Larrey to divide the bridge, and cut the bottom of the wound, where he found several nervous bridles. This operation was very painful; but two hours afterwards the patient was much relieved, and in the space of two days all the symptoms disappeared.

"The second was that of a man who received an injury from a spear on the right side of the forehead. The point of the spear had slided obliquely from below upwards and inwards under the pericranium, so as to make a deep fissure in the frontal bone: one of the superciliary nerves was grazed by the cutting side of the spear. Nine days passed without any alarming symptoms, and it was considered as a simple wound; but in the night between the ninth and tenth days tetanus came on, with convulsive motions of the corresponding eye-lids, and a loss of sight in that eye: there was a little mental wandering, a very acute pain, locked jaw, and a very marked disposition to emprosthotonos. Emollients were immediately applied to the wound, and diaphoretic and opiate draughts were given without effect; the complaints went on increasing, and in twenty-four hours would have been at their greatest height. The wound was then examined with a probe, which gave very acute pain; this determined Larrey to divide from below upwards with a bistoury the whole of the superciliary muscle, the nerves, and vessels: the patient was immediately relieved, and in less than twenty-four hours all the tetanic symptoms had disappeared." 345.

Mr. Swan hints his method of treatment with diffident suspicion. He advises general and local blood-letting—calomel with purgatives to restore the secretions—and such medicines as Dover's powder afterwards. It is much to be lamented that, however plausible such remedies may seem, experience, the rigid judge of their real value, has already tried, and found them wanting.

Here, then, we part from Mr. Swan. We think he is deficient in the useful, indeed the necessary, qualities of method and of condensation. We trust that the next edition will appear with emendations, corrections, and reductions. It contains many valuable, and some unimportant facts—some ingenious, and some rather trivial reasoning. We have endeavoured to select the better portions, and have avoided, as much as possible, the occasion for criticism. Again, we would recommend Mr. Swan to follow old Selden's advice, and employ his time in making his book shorter.

